# Effects of Forest-Based Interventions on Mental Health: A Meta-Analysis of Randomized Controlled Trials

**DOI:** 10.3390/ijerph19084884

**Published:** 2022-04-17

**Authors:** Mi-Jung Kang, Hyun-Sun Kim, Ji-Yeon Kim

**Affiliations:** College of Nursing, Eulji University, 712, Dongil-ro, Uijeongbu-si 11759, Korea; gippem@eulji.ac.kr

**Keywords:** forest therapy, meta-analysis, mental health, depression, anxiety

## Abstract

Forest-based interventions are a promising alternative therapy for enhancing mental health. The current study investigated the effects of forest therapy on anxiety, depression, and negative and positive mental condition through a meta-analysis of recent randomized controlled trials, using the PRISMA guideline. Of 825 articles retrieved from databases including PubMed, EMBASE, CINAHL, Cochrane, and PsycINFO, 6 met the inclusion criteria. The results of this study showed that forest-based interventions improved the mental health of participants in the intervention groups when compared to those in the control groups. Thirty-four outcome variables were analyzed from six studies. The overall effect size of the forest therapy programs was 1.25 (95% CI = 0.93–1.57, *p* < 0.001), which was large and statistically significant. These findings imply that forest-based interventions can improve mental health as a nonpharmacological intervention. This study is significant in that it is a meta-analysis of mental health that included only high-quality domestic and international RCTs. In future studies, more RCTs related to various forest interventions and studies involving many participants should be undertaken, which will complement heterogeneity in future meta-analysis studies.

## 1. Introduction

The living spaces of modern people, which have undergone rapid urbanization, have drastically evolved from nature-centered environments to artificial environments. While modern urban development and technological advancement provide convenience and comfort, complex and fragmented urban environments can cause increased stress and exacerbate various chronic health problems [1,2]. Against this backdrop, those who are stressed by city life have explored various stress-relief measures, and seeking relaxation in a natural environment has been found to be an effective strategy. In particular, forest therapy is gaining popularity as a clinically proven intervention for stress relief and management [3].

Recently, there has been an increasing interest in forest therapy, which promotes the improvement of emotional and psychological health using the natural environment of the forest, and forest therapy’s effects on health and well-being have been studied in various contexts [3,4,5]. In a survey of forest therapy program participants, the major reason for their participation was health improvement, and 83.6% of the participants perceived forest therapy to be effective at promoting health or curing diseases [6].

Due to the growing interest and demand for forest therapy, some governments have enacted corresponding laws. Recognizing the health promotion functions of forests, forest therapy is defined as an activity that enhances the immune system and health using various elements from nature, such as scents and scenery [7]. In response to these measures, the Korean government began to create healing forests and has continued to expand the project on the national level [6,8]. As this project has expanded, studies have been conducted on the disease management effect of forest therapy programs, sparking interest among not only the general public, but also people with conditions that may benefit from these programs [4].

The methods applied in forest therapy and prevention programs vary considerably. The core of forest-based programs consists of the use of the five senses (sight, smell, hearing, touch, and taste) to experience the forest, accompanied by activities such as meditation, walks, hiking, videos, and cognitive behavioral therapy [9]. In previous studies, forest walking programs had a positive effect on physiological factors such as blood pressure, lung function, and immune system markers such as interleukin and lymphocyte [10,11]. Other studies also reported the positive effects of forest therapy programs involving forest bathing, walking, and drug treatments on physical symptoms such as blood pressure, heart function, and inflammation level (high-sensitivity C-reactive protein, hs-CRP), as well as mental health [12,13,14]. These results have demonstrated the effects of forest therapy combined with various activities on physical, psychological, and mental health.

Among all of forest therapy’s effects, the psychological effect of forest therapy has received special attention since people who live in urban environments are at an increased risk of long-term exposure to stressful situations and mental health problems [1]. A forest therapy program that included forest folk dancing and forest-based exercises including walking and stretching had significant effects on mood and stress relief [15]. Forest therapy involving meditation and exercise significantly decreased depression in people with alcoholism [16]. Forest walking programs have also been found to lower distress, increase mindfulness [17], and significantly improve quality of life [12]. Furthermore, recently developed forest therapy programs that incorporate virtual reality and videos were found to be significantly effective at improving subjects’ mood, depression, and sense of restoration in numerous studies on the psychological effects of forests [18,19].

Therefore, this study aimed to conduct a comprehensive and up-to-date overview of randomized controlled trials (RCTs) to understand the effects of forest-based interventions on depression, anxiety, and positive and negative mental condition. In addition, by analyzing the overall effect sizes of the RCTs through a meta-analysis, it was sought to provide scientific evidence that could serve as a basis for the development and application of forest-based interventions in the future.

## 2. Materials and Methods

### 2.1. Study Design

This meta-analysis integrated and analyzed the results of RCTs on forest therapy.

#### Criteria for Literature Selection

This study was conducted in compliance with the systematic review reporting guidelines specified in the Cochrane Handbook for Systematic Reviews of Interventions [20] and the 2020 Preferred Reporting Items for Systematic Reviews and Meta-Analysis statement [21]. For the literature selection, we determined the key questions (participants, intervention, comparisons, outcomes, and study design (PICO-SD)) related to forest therapy and conducted an electronic database search according to the selection and exclusion criteria.

According to the selection criteria, the participants included all subjects, and the intervention was forest therapy. Comparisons were conducted using studies that included no-treatment control groups (participants who did not receive forest therapy), treatment-as-usual groups, and alternative intervention groups. Outcomes were limited to those related to mental health that indicated the results of an intervention. The study design was limited to RCTs. In addition, only academic journal publications and dissertations published in English and Korean were selected. If a published paper was found to be based on a dissertation, only the former was selected for analysis.

The exclusion criteria were as follows: (1) studies with designs other than RCTs, (2) studies that included a combination of various interventions other than forest therapy, therefore restricting a separate assessment of the effects of forest therapy, (3) studies that included a control group that underwent an alternative therapy combined with forest therapy, (4) intervention studies that included a control group but only described the pre-treatment and post-treatment results for a single group, (5) case studies or qualitative studies, including descriptive studies based on interviews, review articles, clinical trials, research studies, and meta-analyses, (6) studies with outcome variables other than mental/psychological outcomes, (7) studies that did not include means and standard deviations of the pre-treatment and post-treatment values for the experimental and control groups, (8) studies written in languages other than English and Korean, and (9) studies in which the original text could not be verified.

### 2.2. Data Collection

From 4 to 6 August 2021, three researchers searched five international search engines to collect data, including The Cochrane Register Controlled Trials (CENTRAL), PubMed, Embase, and Cumulative Indexing Nursing and Allied Health Literature (CINAHL). In addition, after conducting an online search of the databases, the list of references was also searched manually. There were no restrictions related to the publication period, and the languages were limited to English and Korean so that the researchers could interpret and understand the papers.

In PubMed, only RCTs were searched, and the keywords used for the search were forest environment [tiab] OR forest area [tiab] OR shin-rin [tiab] OR shinrin-yoku [tiab] OR shinrinyoku [tiab] OR forest walking [tiab] OR forest treatment [tiab] OR forest healing [tiab] OR forest remedy [tiab] OR forest therapy [tiab] OR forest bathing [tiab] OR forest viewing [tiab] OR phytoncide [tiab] OR forest trip [tiab]. In the rest of the search engines, only clinical trials were searched using the following keywords: forest environment OR forest area OR shin-rin OR shinrin-yoku OR shinrinyoku OR forest walking OR forest treatment OR forest healing OR forest remedy OR forest therapy OR forest bathing OR forest viewing OR phytoncide OR forest trip. A total of 825 articles were selected through this process.

### 2.3. Evaluation of the Quality of Literature

To evaluate the quality of the selected literature, theRisk of Bias (RoB) 2.0, Cochrane, Chichester, UK [20], an evaluation tool to determine the quality of RCTs, was used. The tool uses both the checklist method and the domain evaluation method and consists of five evaluation domains: bias arising from the randomization process, bias due to deviations from the intended interventions, bias due to missing outcome data, bias in the measurement of outcomes, and bias in the selection of reported results. The items in each domain are rated according to the RoB as “yes”, “no”, “probably yes”, “probably no”, and “no information”. Next, the final RoB for each domain was determined using the evaluation results of the signal questions for each domain according to the algorithm presented in RoB 2.0, resulting in an RoB of “low”, “some concerns”, or “high”. The evaluation of the “bias due to deviation from the intended intervention” domain depends on how the outcome of interest is determined. The effect of the outcome of interest is divided into the intention-to-treat effect (ITT, the effect of assignment to the intervention group at baseline) and the per-protocol effect (PP, the effect of complying with the intervention specified in a trial protocol), and different algorithms are applied accordingly.

Finally, in the assessment of the overall RoB, “low” indicates that the likelihood of bias is low in all domains, and “some concerns” indicates that there are some concerns with regard to the possibility of bias in at least one domain. “High” indicates that there is a high probability of bias in at least one domain. To evaluate the quality of the literature, the three researchers individually analyzed the results, and for items with inconsistent analysis results, the researchers met to review those items again and reach a consensus.

### 2.4. Data Analysis

#### 2.4.1. Data Coding and Processing

To analyze the characteristics of the literature on forest therapy interventions, one of the researchers created a coding manual classified by study design, study type, publication year, characteristics of subjects in the experimental and control groups, interventions for the experimental and control groups, the duration of the intervention, and the measurement tools used. In order to increase the reliability and ensure consistency between the raters, the 3 researchers each used the coding manual to review 29 studies. After coding, abnormal values or inconsistencies in the research data were checked and finalized after a consensus was reached.

The intervention effect was subdivided for the analysis using the categories: overall mental health, depression, anxiety, positive emotions, and negative emotions. Depression, anxiety, and negative emotions were coded using mathematical transformation. The results were shown using pre-treatment and post-treatment means, standard deviations, and the sample sizes of the experimental and control groups. For studies with multiple measurements, the first scores obtained after completion of the intervention were used for analysis to determine the immediate effect.

#### 2.4.2. Effect Size

Stata SE version 13.1 (StataCorp, College Station, TX, USA) was used for statistical analysis to estimate the homogeneity and effect size. To analyze individual studies with different populations, a random-effects model based on the DerSimonian and Laird method was used. A random-effects model estimates the distribution of effect sizes rather than the common effect size for the intervention effects of individual studies, assuming that they follow a normal distribution. Therefore, the differences in effects between studies should be determined while also factoring in the differences between studies and the differences between the sampling error and the true effect [22]. In general, when heterogeneity between studies is high, a random-effects model is recommended since it provides more conservative estimates than a fixed-effects model (Higgins’ I^2^ > 50% or Cochrane’s Q statistics *p* < 0.1) [22].

The direction and confidence interval (CI) of the effect sizes were identified through forest plots for each outcome variable. In cases when the same variables were measured using different measurement tools, the effect sizes were calculated using the mean, standard deviation, and Hedges’ g, which represents the standardized mean difference of pre-treatment and post-treatment values. Hedges’ g values can indicate small (0.2), medium (0.5), and large (0.8) effect sizes [22]. Statistical significance for the effect size was set at 0.05, and 95% CIs were used. In this study, the Higgins I^2^ statistic was used to evaluate heterogeneity, and I^2^ was calculated as follows:

I^2^ = 100% × (*Q − df)/Q* (1)

where *Q* is the Cochrane heterogeneity statistic and *df* is the degree of freedom. Negative I^2^ values were treated as 0, and the I^2^ values ranged from 0% (no heterogeneity) to 100% (maximum heterogeneity). In general, 50% was considered to indicate moderate heterogeneity, and 75% was considered to indicate high heterogeneity [20].

#### 2.4.3. Publication Bias

To assess publication bias, Egger’s linear regression test [23] was used. This statistical test shows the relationship between the effect sizes of individual studies on the standard errors of the intervention effects as a regression equation. The null hypothesis was that the intercept was a result of chance and thus could not prove the existence of publication bias. This test is also known to estimate the actual estimate of the effect size more accurately than the Begg and Mazumdar rank correlation test [24].

## 3. Results

### 3.1. Selection of Literature

**Figure 1:**
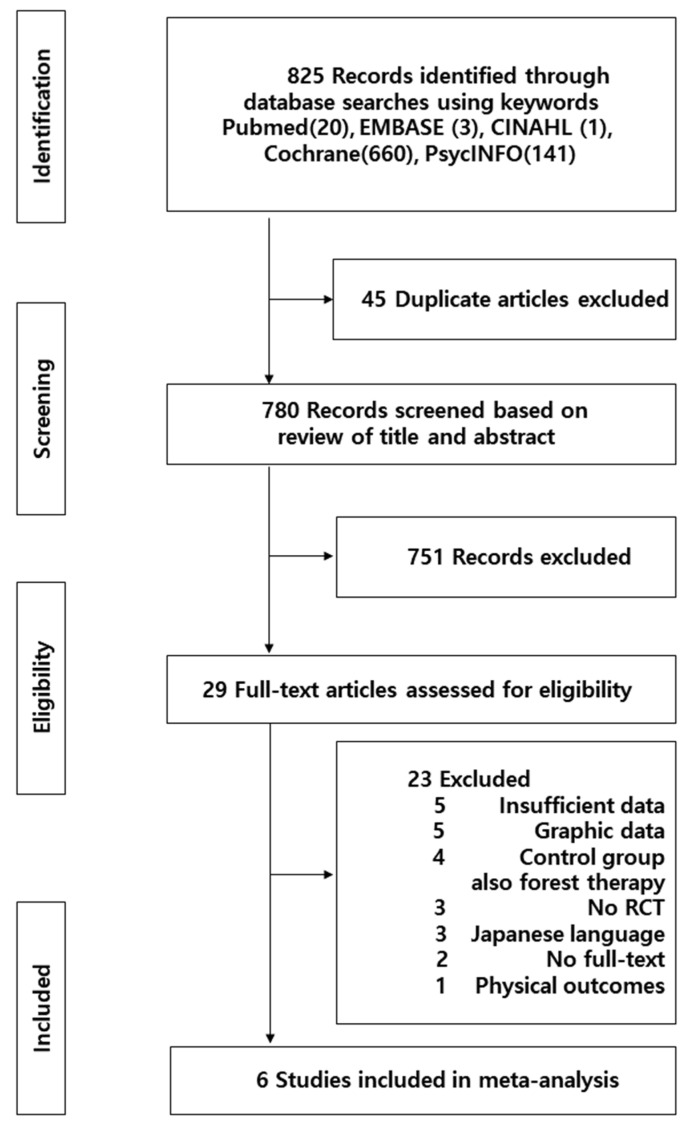
Flow diagram for the study selection process.

The data selection process was as follows. A total of 825 papers were searched using our search strategy for each database (20 from PubMed, 3 from Embase, 1 from CINAHL, 660 from Cochrane, and 141 from PsycINFO). After creating a list of articles from each database, 45 duplicate articles were excluded using EndNote X9 (a bibliographic export program). After reviewing the titles and abstracts of the remaining 780 articles, 751 were removed according to the selection and exclusion criteria, and the remaining 29 original articles were analyzed further. Of the total 29 articles, 6 studies were ultimately included in the analysis after 23 articles were excluded that did not meet the selection criteria for the reasons listed in Figure 1.

### 3.2. Evaluation of the Quality of the Literature

Table 1 shows the results of the quality evaluation of the selected literature. All of the studies used in this analysis investigated the effect of complying with the intervention specified in the trial protocol, and the PP effect algorithm was applied. First, regarding bias arising from the randomization process, five studies (83.3%) had a low RoB, while one study (16.7%) was considered to have possible bias since the experimental intervention and the control intervention were randomly watched and evaluated. The RoB caused by deviations from the intended interventions, including blinding of participants and investigators, was low for all of the studies. Four studies (66.7%) had a low RoB and two studies (33.3%) had a high RoB due to missing data on intervention results. Among the studies with a high RoB, there were differences in the dropout ratio between the experimental group and the control group. In 1 study, 1 subject in the experimental group and 14 subjects in the control group dropped out, while in the other study, 3 subjects in the control group and 12 subjects from the experimental group dropped out. Two studies (33.3%) showed a high RoB and four studies (66.7%) showed a low RoB caused by measurement of the outcome. The RoB was considered high for these two studies since they included subjective self-reported outcome evaluation data and lacked certain information due to an insufficient description of blinding of the outcome evaluators. Bias in the selection of the reported results was low for all studies. Of the six total studies, three studies, all of which had a high RoB in at least one area, were rated as having a high overall risk of bias.

### 3.3. General Characteristics of the Included Studies

A total of six articles were analyzed in this study, all of which were published in academic journals. The largest number of studies (*n* = 3) were written by Korean authors, with 1 study each being written by Italian, Chinese, and Finnish authors. All 6 studies were RCTs published within the last 5 years (4 in 2020, 1 in 2017, and 1 in 2016). Except for one study on chronic stroke patients, all of the study participants were healthy adults (332 in total, with 170 from experimental groups and 162 from control groups). Since some studies did not mention age and sex, we could not conduct an analysis based on these characteristics (Table 1).

The contents of the interventions from four studies included direct experiences of the forest, while two studies included video experiences. The direct experience programs involved forest bathing, in which the participants sat quietly for 2 h, forest therapy programs (forest dancing, forest meditation, forest exercises, and walking), a 4-day and 3-night forest therapy program (meditation, experiencing the forest using the 5 senses, and walking in the forest), and a forest-walking program. For the video-watching programs, the subjects were asked to watch a video containing visual and auditory stimuli that could be experienced in a forest. While there was no treatment control in one study, a control group was asked to watch a video of a city in two studies. One study included a control group that stayed in an urban area in a hotel and participated in meditation and walking activities that were similar to those performed by participants in the forest. Finally, 1 study included a control group that was instructed to sit quietly for 2 h at a suburban location. Only one study did not specify the control group treatment.

The variables used to measure the effects of interventions were depression, anxiety, positive emotions, and negative emotions (Table 1). Depression-related variables were measured using Profile of Mood States (POMS) scores for depression and depression-dejection, Modified Stress Response Inventory (SRI-MF) scores for depression, Beck’s Depression Inventory (BDI), and a 17-item version of the Hamilton Depression Rating Scale (HAM-D17), which were included in five studies. Anxiety-related variables were measured using the Sheehan Patient-Rated Anxiety Scale (SPRAS), State-Trait Anxiety Inventory forms Y1 and Y2 (STAI-Y1, Y2), and POMS scores for tension-anxiety, which were included in five studies. Positive emotion-related variables were measured using POMS scores for vigor-activity and vigor, the restorative outcome scale (ROS), the subjective vitality scale (SVS), and quality of life, which were included in four studies. Finally, negative emotion-related variables were measured using POMS scores for fatigue-inertia, forgetfulness, irritation, slackening, and insecurity, as well as SRI-MF scores for total stress response, somatization, and anger, which were included in two studies (Table 1).

### 3.4. Outcome Variables and the Effect Sizes of Forest Therapy Intervention Studies

**Figure 2:**
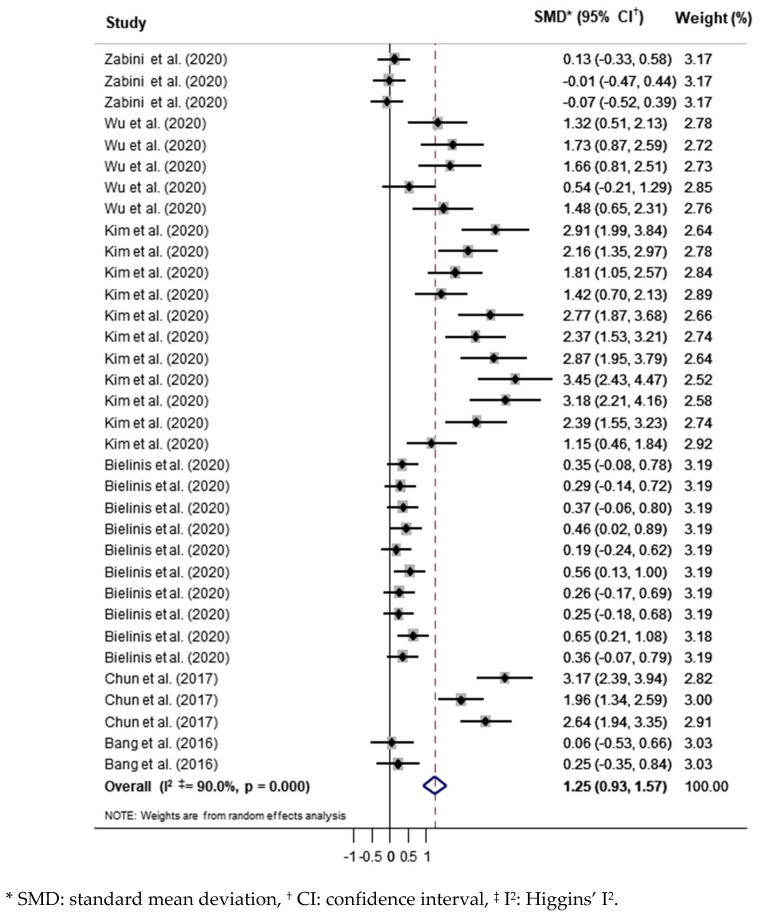
Effects of forest-based interventions on mental health.

In this study, we analyzed a total of 34 outcome variables from 6 studies. The overall effect size of the forest therapy programs was 1.25 (95% CI = 0.93–1.57, *p* < 0.001), which was large and statistically significant (Figure 2). Heterogeneity was high (I^2^ = 90.0%). The results of the analysis of the effect variables were classified into four subgroups (depression, anxiety, positive emotions, and negative emotions).

#### 3.4.1. Depression

**Figure 3:**
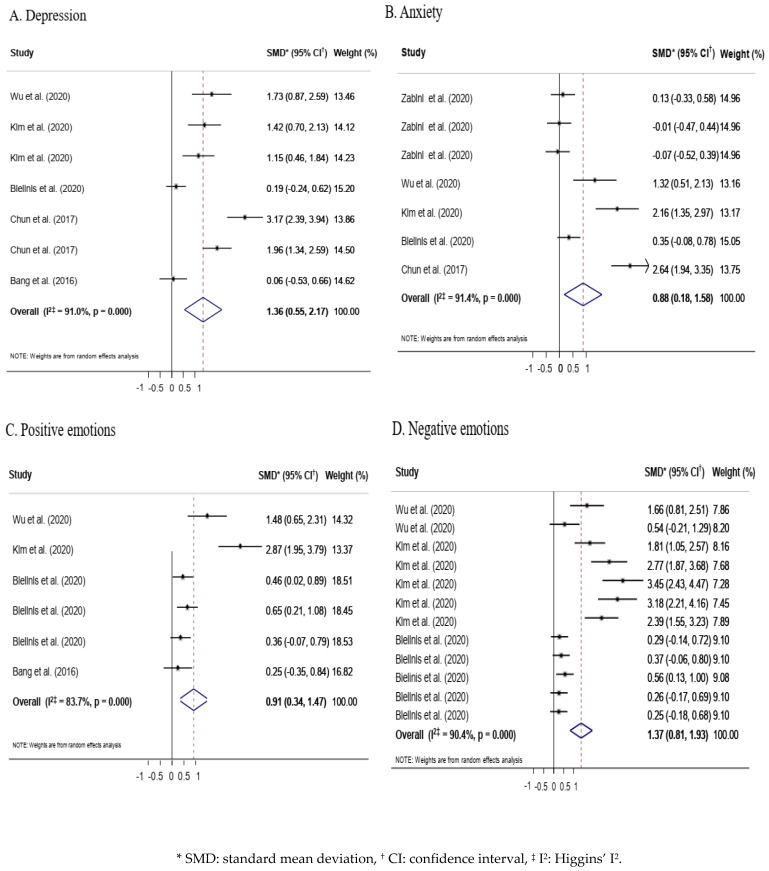
Effects of forest-based intervention on subgroups of mental health.

Seven outcome variables that included POMS scores for depression and depression-dejection, SRI-MF scores for depression, BDI, and the HAM-D17 were extracted from five studies to analyze the effect variables in the depression domain. The overall effect size was 1.36 (95% CI = 0.55–2.17, *p* < 0.001), which was large and statistically significant. Heterogeneity was high at I^2^ = 91.9% (Figure 3).

#### 3.4.2. Anxiety

Anxiety-related variables were measured using the SPRAS, the STAI-Y1/Y2, and POMS scores for tension-anxiety. Seven outcome variables from five studies were analyzed. The effect size was 0.88 (95% CI = 0.18–1.58, *p* < 0.001), which was very large and statistically significant. Heterogeneity was high (I^2^ = 91.4%) (Figure 3).

#### 3.4.3. Positive Emotions

Positive emotions were measured using POMS scores for vigor-activity and vigor, the ROS, the SVS, and quality of life. Six outcome variables extracted from four studies were analyzed. The effect size was 0.91 (95% CI = 0.34–1.47, *p* < 0.001), which was very large and statistically significant. Heterogeneity was high at I^2^ = 83.7% (Figure 3).

#### 3.4.4. Negative Emotions

Negative emotions were measured using POMS scores for fatigue-inertia, forgetfulness, irritation, slackening, and insecurity, as well as SRI-MF scores for total stress response, somatization, and anger. Nine outcome variables from two studies were analyzed. The effect size was 1.37 (95% CI = 0.81–1.93, *p* < 0.001), which was very large and statistically significant. Heterogeneity was high (I^2^ = 90.4%) (Figure 3).

#### 3.4.5. Testing for Publication Bias

The Egger’s linear regression test found a bias of 9.183 (*p* < 0.001). Since the null hypothesis that there would be no correlation between the effect size (*x*-axis) and the standard error (*y*-axis) was rejected, publication bias was confirmed.

## 4. Discussion

This study sought to provide a basis for forest-based interventions as a viable nonpharmacological intervention via a systematic literature review and meta-analysis to gain an in-depth understanding of the effects of forest-based interventions on depression, anxiety, and positive and negative mental health conditions reported in RCTs.

All six RCTs were published within five years of this study, indicating that RCTs have become more common compared to the past, when single-group experimental and nonequivalent-control group designs were predominant. As a result of the analysis of 34 effect sizes from 6 studies, the overall effect size of forest-based interventions on mental health was found to be very high, at 1.25.

Chae and Lee reported in a systematic literature review that studies on the mental health effects of forest-based interventions on adult subjects most often focused on depression, which was found to be significantly reduced in the experimental group [25]. The next most common subject of focus was anxiety, which also showed a significant decrease after forest-based interventions. In the current study, we also found that previous studies on the effects of forest-based interventions most often focused on depression and anxiety, both of which showed large effect sizes at 1.36 for depression and 0.88 for anxiety. In addition, positive emotions showed an effect size of 0.91 and negative emotions showed an effect size of 1.37, indicating that the effect of forest-based interventions on reducing negative emotions was greater than their effect on enhancing positive emotions. These results correspond to the results of a previous systematic literature review and meta-analysis showing that forest-based interventions had significant effects on stress, depression, anxiety, and negative emotions among mental health factors [5], which supports the results of our study.

Conventional treatment methods for depression and anxiety, for which forest therapy showed the highest effect sizes in the current study, are psychotherapy and treatment with antidepressants [26]. However, since psychotherapy and drug treatment have disadvantages such as secondary effects and side effects, interventions using natural environments have been recommended as nonpharmacological interventions that can mitigate the downsides of traditional methods [16,27]. In particular, forest therapy was found to be more effective than other alternative interventions for alleviating depression and anxiety [28]. It has been confirmed that walking through or looking at the forest induces physiological relaxation, including relaxation of the autonomic nervous system and central nervous system, as well as psychological relaxation, including stress relief [29]. Likewise, forest therapy has been found to reduce depression and anxiety and increase self-esteem by lowering the concentration of the stress hormone cortisol [30,31]. This is also supported by a study that found that forest trekking, including massaging and stretching, was effective at relieving mental anxiety and tension among postmenopausal women by reducing their cortisol levels [32].

Furthermore, the act of concentrating through meditation and breathing in a forest can have a positive effect on depression and anxiety by promoting the recovery process, inspiring satisfaction with one’s physical health, and providing the ability to self-heal by becoming aware of the inner self [33]. In addition, when drug treatment is combined with cognitive behavioral therapy in a forest setting, antidepressants become even more effective, and forest experience programs improve participants’ self-awareness, inclusiveness, conformity, and openness, thereby facilitating psychological stability and reducing anxiety [34,35]. Therefore, forest therapy can be expected to have healing effects without side effects when compared to existing treatment methods, and has strong potential as an alternative treatment option for depression and anxiety.

The variables associated with positive and negative emotions included quality of life, stress, fatigue, and irritability. Given the results of previous studies that found that people felt more comfortable and relaxed and experienced more natural emotions in a forest environment than in an urban environment, our analysis of positive and negative emotions affected by forest therapy is very meaningful [25]. This has also been confirmed by a study that included subjects who participated in a forest-based intervention who cited that they felt “comfortable”, “natural”, and “relaxed”, and that their negative emotions were significantly reduced [36]. This is likely due to the transformation of negative emotions into positive emotions that takes place when a person recognizes his or her negative emotions in a natural setting, allowing the individual to recover his or her self-esteem through the process of inner immersion and enhancing the ability to cope due to immersion in nature [37]. Therefore, in the future, forest therapy should be actively used to control the positive and negative emotions of patients.

There are many countries that recognize the medical effects of forest therapy. Physicians in Scotland were authorized to prescribe nature instead of medicine to their patients beginning in 2018, and they can provide natural prescriptions that relieve the symptoms and increase the happiness levels of patients with high blood pressure, depression, emotional instability, and heart disease. In New Zealand and the United States, forests are used to encourage physical activity and good nutrition, and forest trails are created in green spaces that are operated as “medical paths” or “prescription paths”. Forests not only preserve the natural environment but also contribute to health and emotions and provide psychological effects by emitting beneficial substances such as phytoncides, terpenes, negative ions, and wave emissions [10]. The mechanisms behind the physical effects of forest-based interventions have been investigated in various ways, whereas the mechanisms of forest-based interventions’ effects on mental health have yet to be studied in detail. Therefore, it is necessary to conduct scientific investigations into the mechanisms behind forest-based interventions’ effects on mental health.

As a result of the evaluation of the RoB of the literature included in this study, three of the six studies had a high RoB. The RoB was high in these studies due to missing outcome data. In addition to a lack of information due to insufficient description of the blinding of outcome evaluators, the inclusion of subjective self-reported outcome evaluation data led to a high RoB in the assessment of intervention outcomes. In the future, the research quality should be improved by providing more detailed explanations of the research process, participants, and dropouts, and selecting more objective evaluation methods, as well as continuously promoting a quantitative increase in RCTs.

This study is meaningful since it contained a meta-analysis of mental health that examined only high-quality domestic and international RCTs and excluded other types of studies, such as single-group studies. However, the study was limited since the heterogeneity was too high in the effect size analysis. The high heterogeneity could be attributable to differences in the number of subjects, the duration and frequency of interventions, and the contents of interventions between studies. In addition, the forest therapy was conducted in a variety of settings, such as natural settings, urban forest, video, etc. Biotic and abiotic conditions in these forest environments can substantially differ from natural settings. This study did not analyze the differences. We suggest future studies on the differences for various forest settings. Nevertheless, the results of this study are meaningful in that forest-based interventions were confirmed to be a promising nonpharmacological intervention for treating mental health in the future. More high-quality RCTs should be conducted to confirm the effects of forest-based interventions and develop forest therapy programs using various media.

## 5. Conclusions

This meta-analysis of RCTs related to forest-based interventions examined their effects on various domains of mental health. The results of this study showed that forest-based interventions improved the mental health of participants in the intervention groups when compared to those in the control groups. These results suggest that forest-based interventions can be used as nonpharmacological interventions to improve mental health. However, due to the high heterogeneity found in this study, more RCTs should be conducted and the number of subjects should be expanded. Furthermore, studies that include other methods of participation applicable in non-face-to-face contexts such as virtual reality should be examined in addition to in-person forest-based interventions.

## Figures and Tables

**Table 1:** General characteristics and risk of bias assessment of included randomized controlled trials (*n* = 6).

Study	Country	Ex.	Com.	Experimental program	Comparator program	Outcome	D1	D2	D3	D4	D5	Overall
1. Zabini et al. (2020)	Italy	41	34	Forest Videos	Urban Videos	SPRAS, STAI	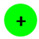	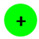	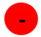	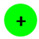	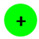	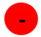
2. Wu et al. (2020)	China	20	11	forest bathing	sit quietly on suburban site	POMS	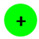	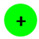	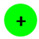	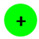	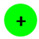	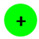
3. Kim et al. (2020)	Korea	19	19	forest therapy program (forest dance, forest meditation, forest exercise, walking)	no description	POMS, SRI-MF	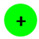	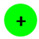	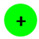	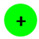	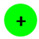	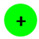
4. Bielinis et al. (2020)	Finland	42	42	Forest Videos	Urban Videos	POMS, ROS, SVS	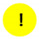	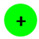	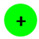	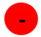	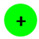	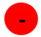
5. Chun et al. (2017)	Korea	30	29	forest therapy program consisted of promoting positive emotion through meditation, experiencing the forest through all five senses and walking in the forest	The urban group stayed in a hotel. The meditation and walking activities were similarly performed in the urban area and the forest	BDI, HAM-D17, STAI	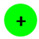	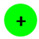	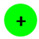	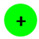	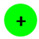	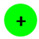
6. Bang et al. (2016)	Korea	18	27	Forest-walking Program	no treatment	BDI, QOL	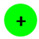	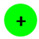	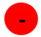	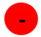	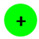	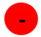

SPRAS: Sheehan Patient Rated Anxiety Scale, STAI: State-Trait Anxiety Inventory Form, POMS: Profile of mood states, SRI-MF: Modified form of the Stress Response Inventory, ROS: restorative outcome scale, SVS: subjective vitality scale, BDI: Beck Depression Inventory, HAM-D17: 17-item version of the Hamilton Depression Rating Scale, QOL: Quality of life, Ex.: Experimental sample size, Com.: Comparator sample size. 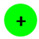: Low risk, 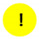: Some concerns, 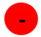: High risk. D1: Randomisation process, D2: Deviations from the intended interventions, D3: Missing outcome data, D4: Measurement of the outcome, D5: Selection of the reported result.

## Data Availability

Not applicable.
